# Surgical management of Wilkie syndrome in young patients: a systematic review and the first report of long-term endoscopic follow-up

**DOI:** 10.1093/jscr/rjaf939

**Published:** 2025-11-25

**Authors:** Gabriela Boff Comiran, Marcelo Faedo Turra, Gustavo Smaniotto Bruckchen, Felipe Hamann Baptista, Luiza Dieguez Piber, Ana Carolina Chiesa Ferri, Leonardo Santini Pedrosa da Fonseca, Luiz Henrique Bastos Capaverde

**Affiliations:** School of Medicine, Pontifical Catholic University of Rio Grande do Sul (PUCRS), Av. Ipiranga, 6681 - School of Medicine - 12A, Porto Alegre, Rio Grande do Sul, CEP 90619-900, Brazil; General Surgery Residency Program, Hospital São Lucas, Pontifical Catholic University of Rio Grande do Sul (PUCRS), Av. Ipiranga, 6681 - Faculdade de Medicina - 12A, Porto Alegre, Rio Grande do Sul, CEP 90619-900, Brasil; School of Medicine, Pontifical Catholic University of Rio Grande do Sul (PUCRS), Av. Ipiranga, 6681 - School of Medicine - 12A, Porto Alegre, Rio Grande do Sul, CEP 90619-900, Brazil; School of Medicine, Pontifical Catholic University of Rio Grande do Sul (PUCRS), Av. Ipiranga, 6681 - School of Medicine - 12A, Porto Alegre, Rio Grande do Sul, CEP 90619-900, Brazil; School of Medicine, Pontifical Catholic University of Rio Grande do Sul (PUCRS), Av. Ipiranga, 6681 - School of Medicine - 12A, Porto Alegre, Rio Grande do Sul, CEP 90619-900, Brazil; School of Medicine, Pontifical Catholic University of Rio Grande do Sul (PUCRS), Av. Ipiranga, 6681 - School of Medicine - 12A, Porto Alegre, Rio Grande do Sul, CEP 90619-900, Brazil; General Surgery Residency Program, Hospital São Lucas, Pontifical Catholic University of Rio Grande do Sul (PUCRS), Av. Ipiranga, 6681 - Faculdade de Medicina - 12A, Porto Alegre, Rio Grande do Sul, CEP 90619-900, Brasil; Department of Surgery, School of Medicine, Pontifical Catholic University of Rio Grande do Sul (PUCRS), Av. Ipiranga, 6681 - School of Medicine - 12A, Porto Alegre, Rio Grande do Sul, CEP 90619-900, Brazil

**Keywords:** Wilkie syndrome, superior mesenteric artery syndrome, laparoscopic duodenojejunostomy, endoscopic, case report

## Abstract

Wilkie syndrome, or superior mesenteric artery syndrome (SMAS), is a rare cause of upper gastrointestinal obstruction that predominantly affects young patients with low body mass index and presents with nonspecific symptoms. We report the case of an 18-year-old female who underwent laparoscopic duodenojejunostomy after failure of conservative management. The patient achieved complete resolution of symptoms, and long-term endoscopic follow-up confirmed a well-healed duodenojejunal anastomosis. This report highlights the role of surgical management in refractory SMAS and provides the first long-term endoscopic documentation of the postoperative outcome, along with systematic review of the current literature.

## Introduction

Superior mesenteric artery syndrome (SMAS), also known as Wilkie’s syndrome, is a rare cause of upper intestinal obstruction resulting from compression of the third portion of the duodenum between the abdominal aorta and the superior mesenteric artery (SMA). It is often associated with rapid weight loss, anatomical predisposition, or spinal deformities such as scoliosis correction surgery [1–3].

Although SMAS can occur at any age, it predominantly affects adolescents and young adults, especially females, and is frequently associated with low body mass index [4–6]. Clinical presentation is nonspecific, with postprandial abdominal pain, nausea, vomiting, early satiety, and progressive weight loss being the most common findings [7–9].

Diagnosis relies on imaging such as contrast-enhanced computed tomography (CT), magnetic resonance angiography, and upper gastrointestinal endoscopy, which demonstrate duodenal compression and exclude other causes [10, 11]. Initial treatment is conservative, focused on nutritional support and weight restoration. However, when the symptoms persist, surgical intervention becomes necessary, with laparoscopic duodenojejunostomy considered the gold standard [12].

We report the case of a young female patient successfully treated with laparoscopic duodenojejunostomy and, to our knowledge, the first long-term postoperative endoscopic documentation of duodenojejunal anastomosis in SMAS.

## Case report

An 18-year-old female presented with progressive postprandial abdominal pain, early satiety, nausea, and a 7-kg weight loss over two months. Her body mass index was 16.8 kg/m^2^. Physical examination was unremarkable. Initial laboratory findings were within normal limits.

**Figure 1:**
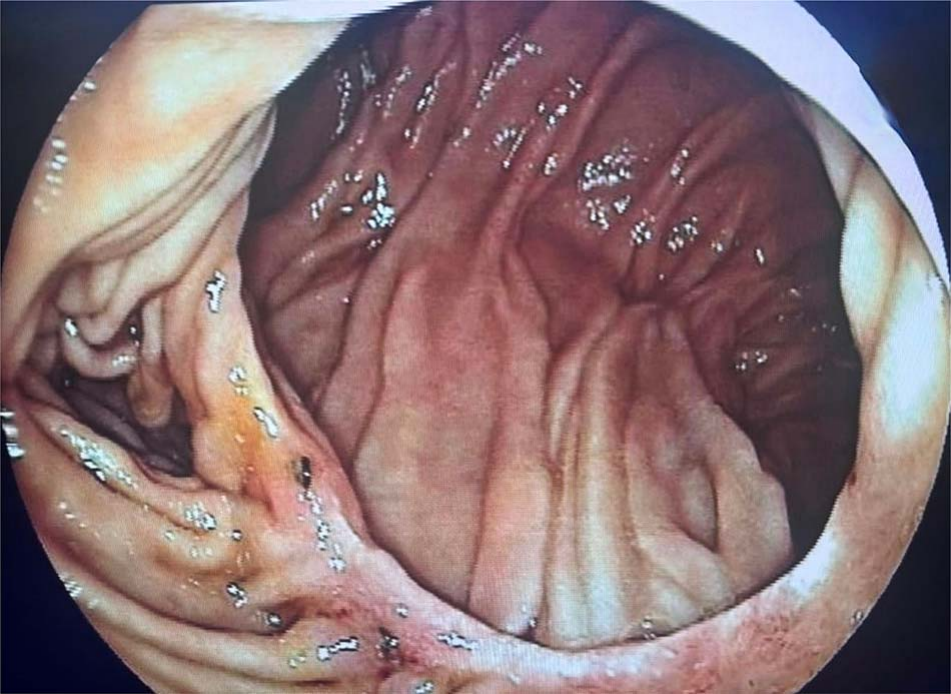
Contrast-enhanced abdominal CT demonstrating compression of the third portion of the duodenum: (A) axial view showing duodenal dilatation and abrupt narrowing; (B) sagittal reconstruction revealing a reduced aortomesenteric angle of 17°.

Contrast-enhanced abdominal CT with angiographic reconstruction demonstrated narrowing of the aortomesenteric angle to 17°, resulting in compression of the third portion of the duodenum (Fig. 1). Upper gastrointestinal endoscopy showed extrinsic pulsatile compression of the third duodenal portion, with no mucosal abnormalities. Based on these findings, a diagnosis of SMAS was confirmed.

**Figure 2:**
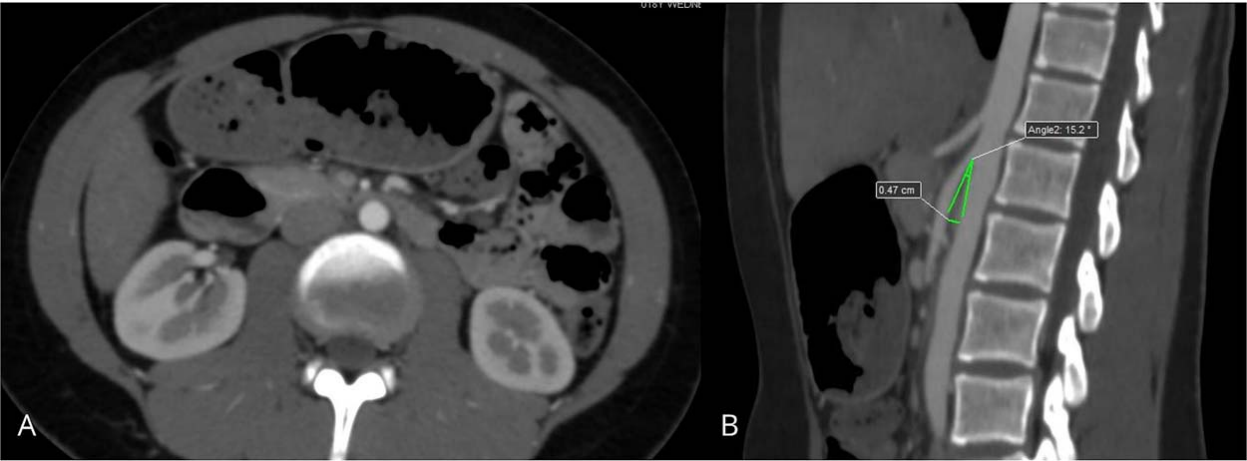
Intraoperative laparoscopic view showing the creation of a side-to-side duodenojejunal anastomosis using a 45-mm linear stapler.

After failure of conservative treatment, the patient underwent laparoscopic duodenojejunostomy in January 2024, performed by a specialized digestive surgery team. The procedure included mobilization of the duodenum and creation of a side-to-side duodenojejunal anastomosis with a 45-mm linear stapler (Fig. 2). Postoperative recovery was uneventful, and the patient was discharged on postoperative day 4.

**Figure 3:**
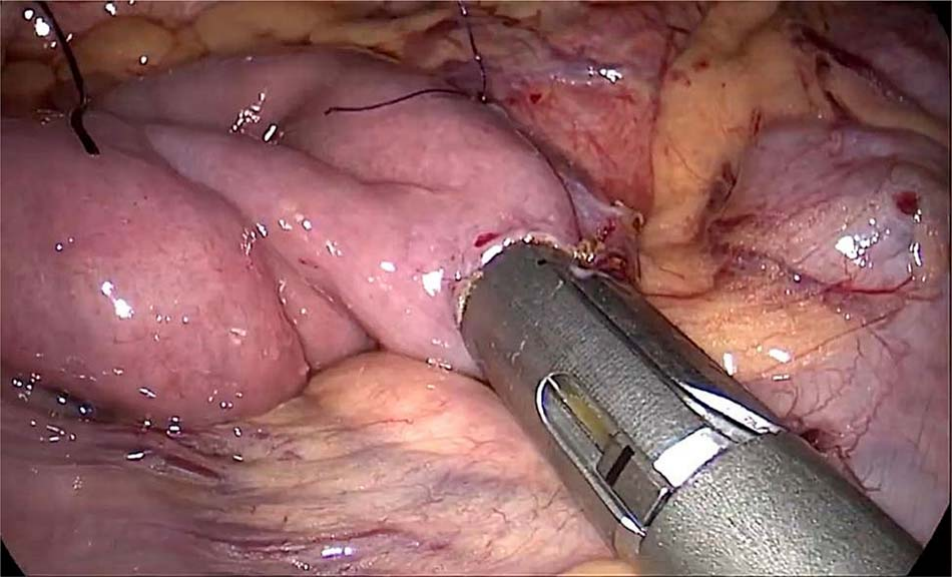
Upper gastrointestinal endoscopy performed one year after surgery demonstrating a widely patent and well-healed duodenojejunal anastomosis without stricture, ulceration, or stasis.

At follow-up, she reported complete resolution of symptoms and progressive weight gain. An upper gastrointestinal endoscopy performed one year after surgery demonstrated a widely patent and well-healed duodenojejunal anastomosis, without signs of stricture, ulceration, or stasis (Fig. 3). To the best of our knowledge, this represents the first published endoscopic documentation of long-term postoperative findings following laparoscopic duodenojejunostomy for SMAS.

## Discussion

SMAS is an uncommon and often underrecognized condition, most frequently affecting young women. In our synthesis of recent literature, the majority of reported patients were adolescents or young adults presenting with postprandial abdominal pain, nausea, vomiting, and significant weight loss. Contrast-enhanced CT was the most widely used diagnostic tool, while endoscopy proved valuable in confirming extrinsic duodenal compression.

Although conservative therapy remains the first-line approach, its success is limited in patients with long-standing or severe symptoms. Across the reviewed reports, laparoscopic duodenojejunostomy was the most frequently adopted surgical option, consistently associated with symptom resolution and nutritional recovery. These findings reinforce the role of surgical management as a definitive strategy when conservative measures fail.

Our case adds a novel contribution by documenting, for the first time, long-term endoscopic follow-up of a duodenojejunal anastomosis after laparoscopic duodenojejunostomy. The postoperative endoscopy, performed one year after surgery, demonstrated a widely patent and well-healed anastomosis without stenosis, ulceration, or stasis. This observation supports the durability of the laparoscopic approach and highlights the potential role of endoscopic evaluation in postoperative monitoring of selected patients.

In conclusion, laparoscopic duodenojejunostomy remains the preferred surgical strategy for refractory SMAS. Our case demonstrates excellent long-term clinical and morphological outcomes and contributes unique endoscopic evidence to the existing literature.
